# The Book World of Medicine and Science

**Published:** 1898-09-03

**Authors:** 


### THE BOOK WORLD OF MEDICINE AND SCIENCE.

LIPPINCOTT'S POCKET MEDICAL DICTIONARY. Edited by RYLAND W. GREENE, A.B., Editor of "Lippincott's Medical Dictionary." (Philadelphia and London. J. B. Lippincott Co. 1897.)

This is a very useful book to lie on the desk of a medical man. If anyone really requires a dictionary which will go into the pocket this is exactly the thing for him, but as a matter of fact few people want to put books into their pockets; what they want is a volume which shall occupy such little space that it can lie on the desk without cumbering it, and thus enable them to have a dictionary always at hand without having to lift from its shelf a ponderous tome. This dictionary is the same size and shape as, and only slightly thicker than, an ordinary pocket visiting list, yet it contains 421 pages of printed matter, giving the definition and, in many cases, the pronunciation of 20,000 of the principal terms used in medicine and the allied sciences. Of course, the definitions are somewhat shorter and the words are somewhat fewer than in the splendid volume produced by the same editor, under the title of "Lippincott's Medical Dictionary," but it is evidently compiled with the same care. The book is well up to date, and contains definitions of a very large number of those very puzzling anatomical and medical terms which are compounded out of proper names. To take one page which happens to be open before us, we find the following derivatives of proper names: Houston's muscle, H.'s valves, Howship's lacunæ, Huguier's canal, H.'s glands, Hunter's canal, Hunyadi Janos water, Huschke's canal, Hutchinson's teeth, Huxham's tincture, Huxley's layer. This is enough to show the help which such a dictionary may be both to reader and writer. Moreover the hints on pronunciation, although not very obtrusive, will be found useful by many. Of course there are plenty of superior people who affect to laugh at dictionary pronunciation, but ordinary men are often thankful enough to be able to make sure whether a vowel is long or short, or a consonant hard or soft.

THE ORIGIN AND SPREAD OF PANDEMIC DIPHTHERIA. By ARTHUR NEWSHOLME, M.D. Lond., &c. (London: Swan Sonnenschein and Co. 1898. Pp. 195. Price 7/6 net.)

This book embodies the result of a vast amount of statistical research into the yearly mortality from diphtheria during the last twenty-five years in almost every civilised region of the globe. Dr. Newsholme's reputation as a statistician in matters of public health is sufficient guarantee of the completeness and accuracy with which this research has been carried out. But, as Dr. Newsholme himself says, since statistical investigation based on data of varying degrees of accuracy is so apt to be received with incredulity, and so many are ready to say "anything can be proved by statistics," it is worth remembering that without statistics nothing can be proved. The figures obtained by Dr. Newsholme have been carefully plotted out in simple diagrams, but they cover so vast a ground—the records of every chief town in England, continental Europe, the United States of America, Australia and New Zealand—that detailed criticism is impracticable in this place. But the author's conclusions—and it is in them that the interest of the book chiefly lies—are in brief two: (1) Diphtheria only becomes epidemic in years when rainfall is deficient, and the epidemics are on the largest scale when three or more years of deficient rainfall immediately follow one another. (2) Dry years imply low ground water, and in the years of epidemic diphtheria the ground water is exceptionally low. As a corollary we have the hypothesis that the specific micro-organism of diphtheria has a double cycle of existence, one phase of which is passed in the soil. The two main propositions are undoubtedly supported by the statistics brought forward; the corollary, perhaps, requires bacteriological support, which, however, Dr. Newsholme does not supply. Logically, Dr. Newsholme is right in his insistence in the case of diphtheria (as Pettenköfer in the case of cholera) on the importance of climatic and seasonal influences in the production of epizootic disease. It is well that at a time like the present it should be so forcibly brought before the profession that epidemic disease is the resultant of more than purely microbic factors. As Sir Edward Fry has said, there is no physical or logical distinction between principal and minor causes or between causes and conditions in the case of two or more constituent parts of a cause, each of which is necessary and none of which is by itself sufficient. Apart altogether from the academical interest it will excite, Dr. Newsholme's theory, if found to be proved, cannot fail to have great influence on the action of sanitary authorities when grappling with diphtheria. We hope, therefore, that it will be examined with no less care, ability, and impartiality than its author has bestowed in the preparation of this book.

PHYSIOLOGY: EXPERIMENTAL AND DESCRIPTIVE. By BUEL P. COLTON, Professor of Natural Science in the Illinois State University. (London: The Scientific Press. 1898. Pp. 423. Illustrated. Price 6s.)

This book is not suitable—nor is it intended to be—for the use of medical students. It is rather, we gather, written as a text-book for use in secondary schools and classes of popular instruction. As such it will doubtless enjoy considerable vogue. The titular use of the word experimental is perhaps a little misleading, inasmuch as the experiments described in the text are rather of the order of crossing fingers on a marble and breathing into lime-water than such as we are accustomed to associate with the term "experimental physiology." The purely physiological part of this work is of the scope, and to some extent on the plan, of Huxley's classical primer. That is to say, no mention is made of that particular series of facts which, above all others, it is desirable that the young adult should learn through a scientific channel. But scattered through the pages are many hints, homely, but none the less valuable, on the general care of health, on domestic hygiene, the use of stimulants, and kindred topics. An elaborate table of poisons and antidotes, and some directions for First Aid are also inserted. The method of artificial respiration advised, however, is one we should imagine neither so simple nor so efficacious as Sylvester's, Marshall Hall's, or Howard's. Not the least valuable portion of Professor Colton's book is given as an appendix, and consists of lengthy extracts from a prize essay (by Mrs. A. Hickmann) on "Food and Cooking." This essay gives, in a pleasant style spiced by much American piquancy, excellent and practical advice, and should be studied by every middle and working-class housekeeper. On the whole this book, though somewhat discursive, is well written; the details are accurate, and the advice is wholesome. The author has, within the limits he has imposed on himself, been undoubtedly successful.

